# Phenotypic heterogeneity in *Bradyrhizobium diazoefficiens* USDA 110

**DOI:** 10.21203/rs.3.rs-5754266/v1

**Published:** 2025-03-20

**Authors:** Sukhvir K. Sarao, Armaan K Sandhu, Ryan L. Hanson, Tanvi Govil, Volker S. Brözel

**Affiliations:** 1 Department of Biology and Microbiology, South Dakota State University, Brookings, USA; 2 Karen M. Swindler Department of Chemical and Biological Engineering, South Dakota Mines, Rapid City, USA, 57701; 3 Department of Biochemistry, Genetics and Microbiology; Forestry and Agricultural Biotechnology Institute (FABI), University of Pretoria, Pretoria, South Africa

**Keywords:** Phenotypic heterogeneity, Soybean, *Bradyrhizobium diazoefficiens* USDA 110, Proteomics, Bet hedging, Soil, Density gradient centrifugation

## Abstract

Bacteria growing in liquid culture are assumed to be homogenous in phenotype. Characterization of individual cells shows that some clonal cultures contain more than one phenotype. Bacteria appear to employ bet hedging where various phenotypes help the species survive through environmental fluctuations. We asked whether the agriculturally significant bacterium *Bradyrhizobium diazoefficiens* USDA 110, which fixes nitrogen with soybean plants, displays phenotypic heterogeneity. We employed Percoll^™^ density gradient centrifugation to separate clonal populations of exponential and stationary phase *B. diazoefficiens* into four fractions and characterized their phenotype by proteomics. Specific phenotypes were then characterized in detail. Fractions varied by cell size, PHA content, lectin binding profile, growth rate, cellular ATP, chemotaxis, and respiration activity. Phenotypes were not heritable because the specific buoyant densities of fractions equilibrated within 10 generations. We propose that heterogeneity helps slow growing *B. diazoefficiens* proliferate and maintain populations in the different environments in soil and the rhizosphere.

## Introduction

*Bradyrhizobium* is an important and widely distributed soil bacterium that forms symbiotic association with legumes such as soybean and fixes atmospheric nitrogen. *Bradyrhizobium* have been reported in large numbers in soils across the world even in locations without leguminous plants ^1^. Even in soybean fields, the majority of *Bradyrhizobium* in soil are never exposed to soybean roots. For long term population success, *Bradyrhizobium* cannot therefore rely on nodulation but must be able to grow and survive in bulk soil. We recently found a diversity of *Bradyrhizobium* species in bulk soil distant to soybean roots, with similar species distribution in soybean rhizosphere ^2^. The crosstalk between the bacterium and host soybean is very specific, and these interactions determine that only specific *Bradyrhizobium* strains form nodules. The non-nodulating *Bradyrhizobium* must persist outside of nodules. We found *B. diazoefficiens, B. elkanii* and *B. japonicum* inside nodules but these species were also well represented in the rhizosphere and bulk soil ^2^. This occurrence of nodulating species in bulk soil far away from soybean roots indicated that they maintain populations independent of roots. Therefore, the population success of *Bradyrhizobium* as a predominant genus in soils indicates that it is able to adapt to multiple environments including nodules, the rhizosphere, and bulk soil where nutrients may be limiting.

Bacterial cells growing in homogenous liquid culture are assumed to be identical. This homogeneity in microbial culture conditions assumes that the physiological conditions such as temperature, pressure, pH, and nutrient availability are uniform, causing the microbial cells throughout the culture to behave the same. An increasing number of studies indicate that bacterial cells do not behave the same, but display more than one set of phenotypes termed phenotypic heterogeneity ^3^. Heterogeneity in populations can be regulated to smaller or larger degrees. Tightly regulated heterogeneity involves a phenotype that is present or absent, such as spore formation in *Bacillus* ^4^, and flagellum expression in virulent *Salmonella* ^5^. Other examples entail a spectrum of outcomes such as the level of nitrogenase in cells of *Klebsiella oxytoca* grown under balanced conditions in a chemostat ^6^.

In select cases a very small proportion of the population displays the heterogeneity, such as in *E.coli* where some cells of antibiotic sensitive populations are slow growing and resistant, and termed persister cells ^7^. In most cases, the phenotype sub-populations are more evenly distributed. For instance, *S. Typhimurium* exhibits phenotypic heterogeneity in expression of the virulence locus type-III secretion system ^8^. *Saccharomyces* populations display heterogeneity in resistance to heat killing ^9^, and also in tolerance to lead ^10^. *E. coli* displays heterogeneity in expression of genes involved in transition to stationary phase ^11^. In addition, both exponential and stationary phase populations displayed a range in buoyant densities as determined by Percoll^™^ density gradient centrifugation. Fractions from the gradient displayed different levels of expression of stationary phase transition genes. Similarly, *S. cerevisiae* displays heterogeneity in buoyant densities ^12^.

Diversification of phenotypes benefits species occurring in heterogenous environments. The different phenotypes in a population may be responsible for bet hedging or division of labor ^13^. Heterogenous expression of type III secretion system in *S. Typhimurium* comes at a fitness cost to cells that engage host epithelial cells, benefitting other cells that do not express the system, an effective division of labor ^8^. Taxa growing in heterogenous environments, or environments where conditions can change are better positioned for long term success through bet-hedging. Each subpopulation would succeed in a different set of conditions. A slow growing soil bacterium such as *Bradyrhizobium* cannot respond quickly to local changes and must have ways to adapt to these conditions and maintain populations until the next cropping season. Thus bet-hedging prepares species for survival under more than one possible set of conditions ^14 15^.

We recently observed surface sugar differences in clonal liquid cultures as the fluorescently labelled lectins SBA (Soybean Agglutinin) and WGA (Wheat Germ Agglutinin) bound differentially ^16 17^. Some cells bound only to SBA, some to WGA, others to both, and many did not bind to either of the lectins, indicating at least four different surface phenotypes present. Members of *Rhizobiaceae* are very specific in their association with lectins produced by plants ^18^. This indicated population heterogeneity for nodulation readiness where only some cells had lectin binding domains, while the remainder needed to rely on other traits to survive. We hypothesized that *B. diazoefficiens* USDA 110 displays phenotypic heterogeneity other than surface sugars. We targeted various stages in batch liquid culture, performed density gradient centrifugation using Percoll^™^, and characterized phenotypes of the fractions obtained. We found that isogenic populations were heterogenous in various phenotypes including buoyant density, cell size, growth rate, polyhydroxyalkanoate (PHA) content, chemotactic ability, and cellular energetics.

## Results

### Populations comprise subpopulations of different buoyant densities

**Figure 1.**
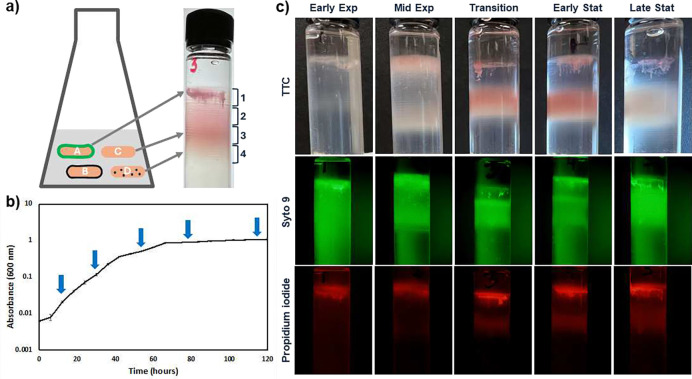
Separation of *B. diazoefficiens* USDA 110 subpopulations by Percoll^™^ density gradient centrifugation. We hypothesized that different phenotypes could be separated by their buoyant densities (a). Sampling time points for early exponential, mid exponential, transition, and early and late stationary phases were determined from the growth curve as indicated by arrows (b). Samples were subjected to density gradient centrifugation after staining with TTC or Syto 9 and propidium iodide (c). Images are representative of experiments performed on four occasions.

Prior work on lectin binding to *B. diazoefficiens* had revealed that only sub populations bound to a specific lectin (Figure S1) ^17^, indicating surface heterogeneity among cells. In a search for approaches to separate heterogenous populations by phenotype, we were inspired by separation of *E. coli* and *Saccharomyces cerevisiae* phenotypes by buoyant density ^11^, ^19^. Buoyant density gradient centrifugation of exponential phase *B. diazoefficiens* revealed many bands with a less defined top domain (Figure 1c), even more bands than visible in *E. coli* ^11^. To determine whether phenotypic heterogeneity extended into stationary phase, we performed a growth curve to define early exponential, mid exponential, transition, early stationary and late stationary (Figure 1b). All growth phases evaluated resolved into many subpopulations, although the distribution of bands differed. Due to the large number of tightly spaced bands, we decided to separate the gradient into four fractions for all further experiments. We termed the top fraction, fraction 1 and the lowest, fraction 4 as shown in figure 1a. Because fraction 1 had the lowest buoyant density, the terms ‘most buoyant’ and ‘upper fraction’ were used interchangeably.

To determine if populations resolved by physiological state, we stained the cultures with TTC and observed presence of color in the various fractions. All fractions appeared pink, indicating respiratory activity. We then resolved cultures stained with Baclight (syto 9 and propidium iodide) to see whether there were any dead cells. To view green and red fluorescence we photographed the same tubes at same magnification. The red fluorescence in the upper less defined top domain pointed to dead cells. Intense green fluorescence at the same locations questioned whether these cells were dead, or whether they were associated with eDNA. Microscopy of this upper domain showed that cells were entangled in clusters similar to as reported in *P. aeruginosa* ^20^. To confirm presence of eDNA we loaded fractions into wells of an agarose gel, but did not observe any fluorescence in the gel (Figure S2). This indicated that *B. diazoefficiens* did not produce eDNA at any of the growth phases in the medium used. Therefore, we concluded that the red fluorescence in the upper domain reflected dead cells. Thus, the upper domain contained both live and dead cells. These results showed that liquid cultures of *B. diazoefficiens* contain cells of multiple different buoyant densities, indicating different phenotypes.

### Cells with different surface properties can be separated by their buoyant densities

**Figure 2.**
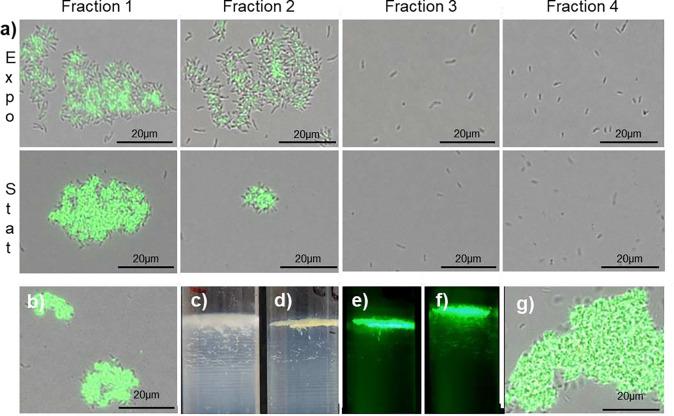
Lectin (Soybean Agglutinin) binding profile of exponential and stationary phase fractions. Fractions exposed to fluorescein conjugated SBA (a) and unseparated exponential populations (b) were viewed by fluorescence microscopy. Gradients of exponential phase populations without (c) and with (d,e) prior treatment with Soybean Agglutinin were viewed by illumination (c,d), and fluorescence (e). Stationary phase populations were pretreated with SBA and viewed by fluorescence (f), and fluorescence microscopy of the S1 showing association of cells by the lectin (g). Images are representative of experiments performed on three occasions

To determine surface sugar differences of various buoyant fractions, we performed lectin binding assay using SBA. The highest proportion of lectin binding cells occurred in E1 and S1, with fewer in E2 and S2, and very few in E3, S3, E4 and S4 (Figure 2a). Most of the cells binding lectin formed clusters. In contrast, cells in the lower fractions did not form clusters. To see whether these clusters are formed during ultra centrifugation, we performed lectin binding on unseparated cultures. Clusters were observed in these cultures (Figure 2b). We have never observed large clusters in non-lectin-stained *B. diazoefficiens* liquid cultures suggesting that clusters form after exposure to SBA. To test this hypothesis, we added SBA to populations before loading on the gradient. Prior exposure to SBA yielded more compact upper domain (Figure 2c vs d), with the lectin localized to the upper domain (Figure 2e). The same phenomenon was observed for stationary phase populations (Figure 2f). This suggested that SBA has a multivalent nature, cross linking cells into clusters ^21^.

### Proteomics shows that fractions differ in only a few phenotypes

**Figure 3.**
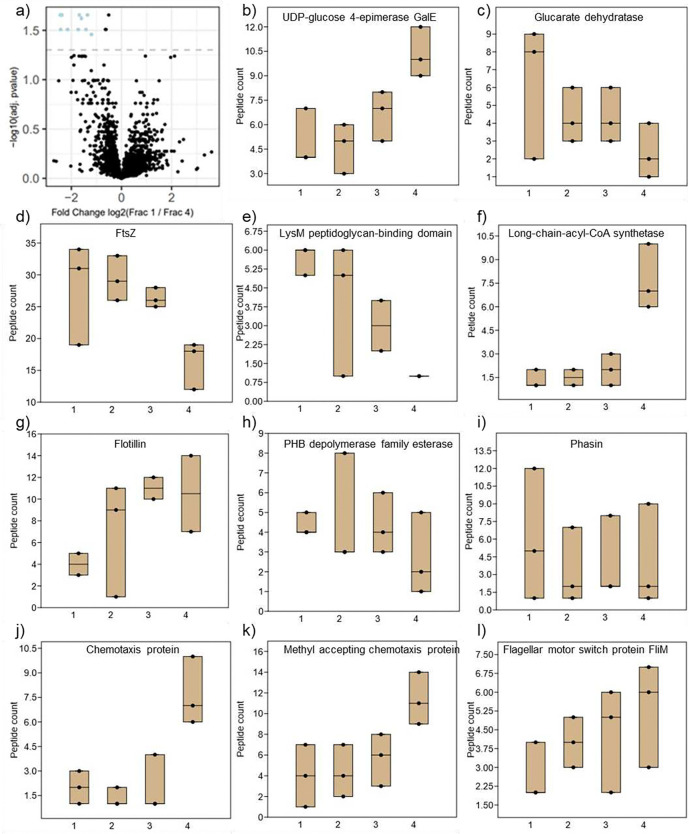
Proteomic characterization of the four exponential phase fractions by LC-MS. Volcano plot comparing E1 and E4, with significant differentially abundant proteins determined using a cut off of 0.05 of adjusted p value at log10 (a). Representative proteins associated with EPS (b,c), Cell envelope and division (d,e,f,g), PHA (h,i), and motility and chemotaxis (j,k,l) with greater than two fold or less than 0.5 are shown. The center lines indicate the median of data from three biological reps.

To obtain an overview of phenotypic differences among subpopulations, we opted for proteomics rather than transcriptomics because bacteria with larger genomes tend to display more post-transcriptional regulation ^22^, and the *B. diazoefficiens* genome is 9.1 Mb ^23^. To identify additional phenotypes unique to the various fractions, we performed proteomic analysis on the four exponential phase fractions. A total of 4594 of the 8283 predicted proteins were detected in exponential phase of *B. diazoefficiens* USDA 110 grown in PSY with arabinose as carbon source. Only 10 proteins were found to be significantly differentially abundant in E1 and E4 as per LFQ analysis in Frag pipe (using a cut off of 0.05 adj. pvalue, 1 log2FC) (Figure 3a). Comparison of E2 and E4 yielded only 8 proteins. Several of these proteins overlapped, with 11 significantly different proteins (Table S1). Comparisons among other fractions showed even fewer significant differences. Because the fractions did not differ much at the proteome level, we did not anticipate a large number of phenotypic differences. To identify potential phenotypic traits for further investigation, we reanalyzed the data to choose proteins that had a ratio between E1 and E4 of either >2 or <0.5. From this list we selected proteins associated with a detectible phenotype, excluding proteins such as transcriptional regulators, putative transporters and alarmones, with selected proteins highlighted in the supplementary spreadsheet. As PHA has been reported to vary among cells of rhizobial populations ^24^, we also selected proteins associated with polyhydroxyalkanoate (PHA) (PHB depolymerase family esterase and Phasin) (Figure 3 h and i).

Proteins involved in polysaccharide synthesis included UDP-glucose 4 epimerase and Glucarate dehydratase (Figure 3b and c). Quantities of EPS in the four exponential phase fractions were near the detection limit (data not shown). EPS quantification of unseparated populations showed that EPS was produced from the transition phase onwards, while exponential phase levels were low (Figure S4). This is analogous to culturing on agar where young colonies are small and later become mucoid. Proteins involved in cell division (FtsZ), peptidoglycan synthesis (LysM peptidoglycan-binding domain) and membrane synthesis (Long-chain-acyl-CoA synthetase, and Flotillin) varied more than two-fold across the fractions (Figure 3 d–g). This prompted us to determine cell size and generation time of the four fractions (see section 4). Proteins involved in chemotaxis (Chemotaxis protein and Methyl accepting chemotaxis protein) and motility (FliM Flagellar motor switch protein) (Figure 3j–l) also varied across fractions. This prompted us to quantify chemotaxis of the four fractions (see section 6).

### Different buoyant density populations have different cell lengths and generation times

**Figure 4.**
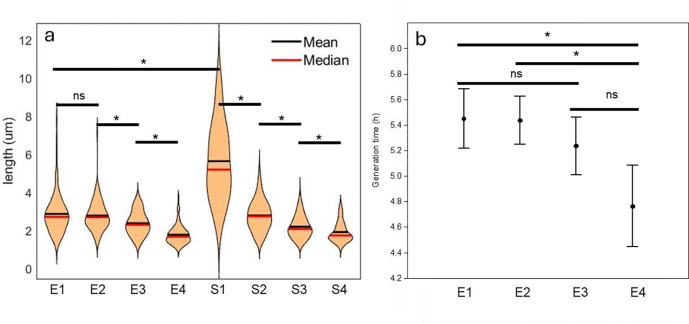
Cell sizes and growth rates of fractions. Exponential and stationary phase fractions differed (a), as did generation times of exponential phase fractions (b). The sizes of all cells in three separate microscopic views were determined, with a minimum of 50 cells. Growth rates were determined using 16 technical reps. The stars signify significant difference (Wilcoxon pairwise p < 0.05).

Cell sizes varied significantly amongst fractions of both exponential and stationary phase populations (Figure 4a). The largest cells had the lowest buoyant density. This suggests that cell length is not proportional to density of cells. Interestingly, S1 contained much longer cells than E1. This is contrary to the established bacterial physiology based on *E.coli* where stationary phase cells are smaller than in exponential phase ^25 26 27^. The observed differences in cell size prompted an investigation into generation times. Plotting of absorbance over time indicated that E4 cells grew faster (Figure S5). Generation times of E4 (4.8 h), the smallest cells were significantly lower than E1 (5.45 h) and 2 (Figure 4b). Again, this is contrary to the relationship between cell size and growth rate in *E.coli* ^28^. Generation times of stationary phase fractions were not determined because the population no longer increases.

### Lowest density has highest proportion of cells with PHA

**Figure 5.**
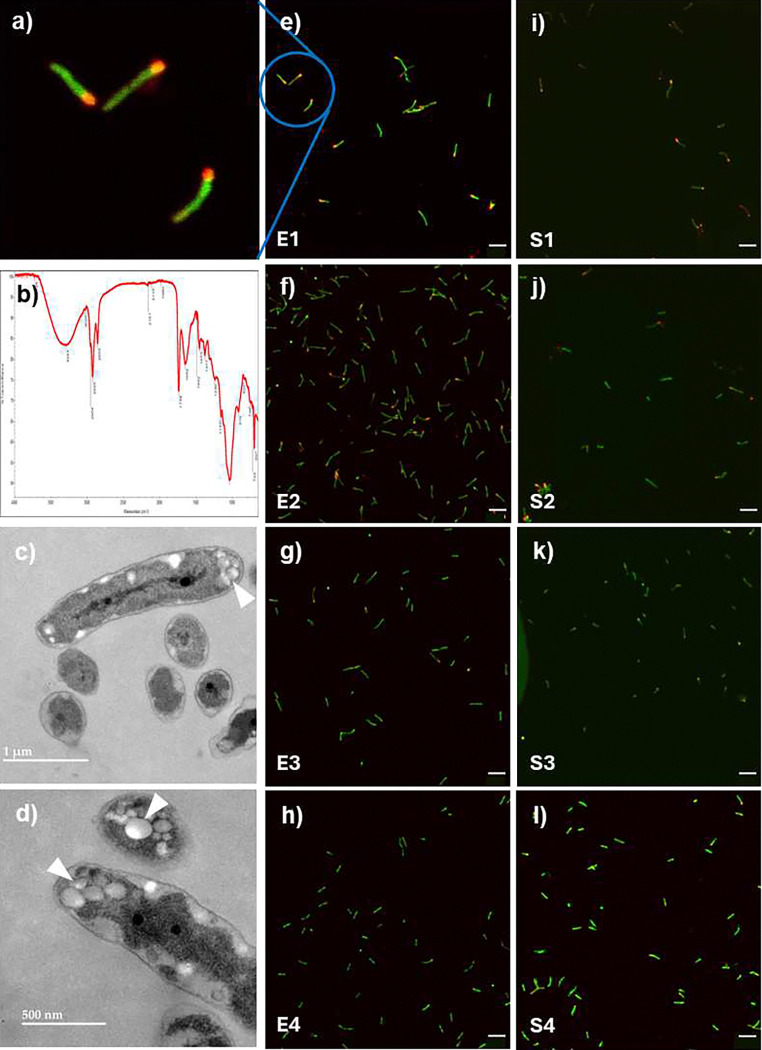
PHA distribution across fractions of exponential and stationary phase populations. PHA was visualized with Nile red and viewed by confocal microscopy (a-i). The presence of PHA was confirmed by the characteristic absorption bands of the extracted material by FTIR spectroscopy (j), and cellular distribution was visualized by Scanning electron microscopy (k,l). Scale bars in e-l represent 5 μm. Images for a and e-l are representative of experiments performed on three occasions, b on one occasion, and c-d on two occasions.

Because PHA has been reported to vary among cells of rhizobial populations, we sought to quantify its distribution amongst cells of various fractions. Almost all the cells in E1 and S1 showed fluorescence with the lipophilic stain Nile Red, known to fluoresce when associated with PHA. The lower fractions had significantly decreased proportions of cells with red fluorescence (Figure 5). Stationary phase populations revealed higher proportions of red fluorescence. The presence of PHA was confirmed by FTIR performed on extracts of exponential phase populations (Figure 5j). Transmission electron microscopy revealed multiple globules of low electron density (Figure 5 k,l). Intriguingly, red fluorescence was only viewed at one of the cell poles as were low electron density granules in TEM, whereas published images of PHA in *B. diazoefficiens* show granules distributed throughout cells ^29 30^. To determine whether polar localization was due to growth in liquid medium versus nodules, or some mutation acquired during culture, we inoculated soybean seedlings with two different strains i.e. *B. diazoefficiens* USDA 110 and *B. diazoefficiens* spc4. Bacteria extracted from the soybean nodules showed PHA granules distributed across the cells (Figure S6). We did not explore this phenomenon further, but these results suggest that PHA localization may differ between liquid grown cells and bacteroids in nodules. Collectively, these results show that more PHA accumulated in E1 and S1 than the other fractions.

The FTIR spectrum of the extract revealed several intense peaks, confirming its identity as PHA. The intense key peak at 1765 cm^−1^ indicates the presence of the ester carbonyl (C=O) stretching, ^31^ which is the predominant bond present in the backbone of PHAs. However, it is important to mention that the carbonyl bond for PHA’s at 1765 cm^−1^ is not the most typical one. The most observed and reported carbonyl bond positions for PHA lie in the region of 1720–1740 cm^−1^. Here, we anticipate that the 1765 cm^−1^ peak could indicate a slight shift may be due to the crystallinity in the PHA molecules, or the presence of additives, unreacted monomers, or solvents from the extraction process. The bands at 2968, 2928, and 2872 cm^−1^ are related to C-H asymmetric resonance of -CH_3_, -CH_2_, and -CH, groups ^32^. The bands at 1223 cm^−1^, 1189 cm^−1^, 1160 cm^−1^, 1101 cm^−1^ indicate the C-O stretching vibrations in the ester group (Alfano et al. 2023); and the series of lower frequency peaks between 1223–1101 cm^−1^ might be indicative of bending or deformation vibrations, including C-H bending and C-O-C stretching in the polymer chain ^33^, ^34^. The broader spectrum peaking at 3282 cm^−1^ is suspected to be the presence of -OH groups, likely due to moisture or water absorbed and remaining in the sample. In our PHA samples, we however also observed some typical bands at 1648 cm^−1^, and 1504 cm^−1^. While the bands at 1648 cm^−1^ could be from residual water (O-H bending) or conjugated C=O groups from PHA degradation products or other impurities), the presence of 1504 cm^−1^ is likely indicative of C=C bonds, possibly from aromatic compounds or unsaturated fatty acids, which may suggest impurities, additives, or contamination.

### Different buoyant densities vary in cell activities and motility

**Figure 6.**
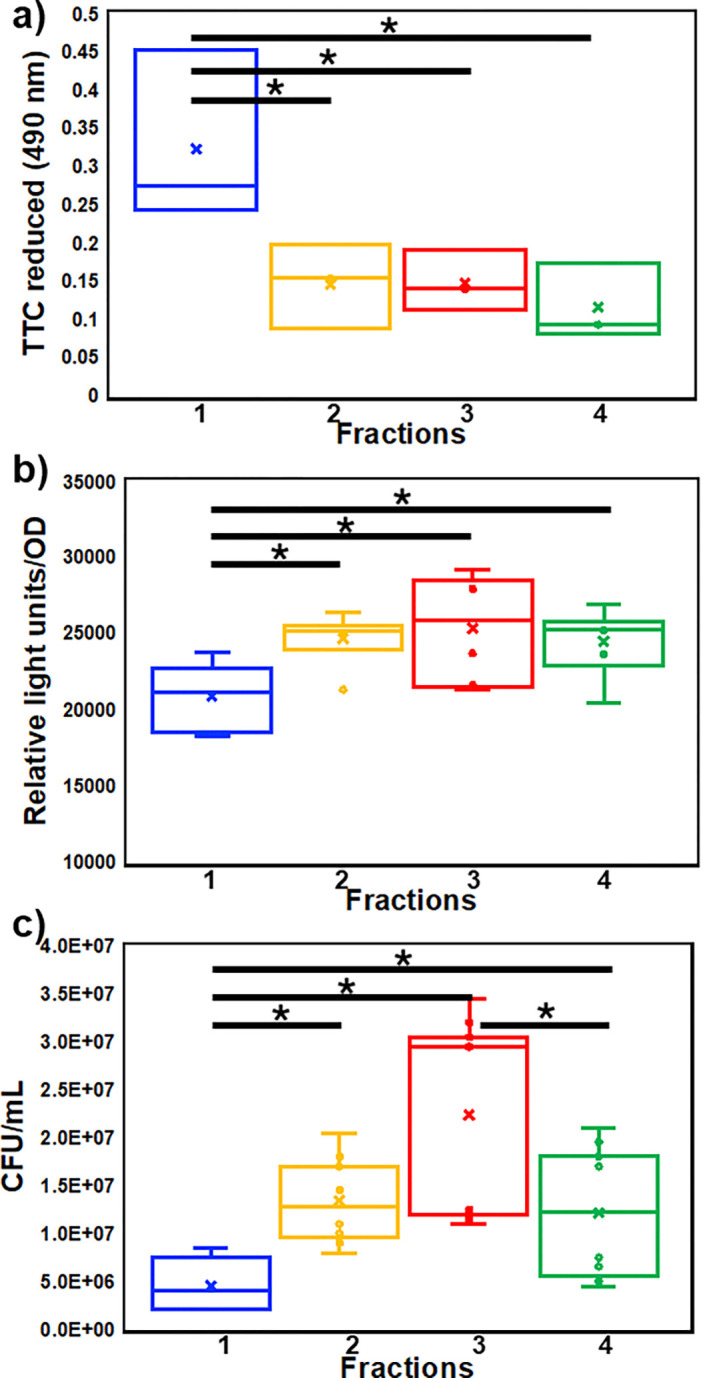
Respiratory activity was quantified by reduction of TTC (Tukey’s pairwise, p < 0.05) (a) and ATP concentration (b) per CFU (Dunn’s post-hoc, p < 0.05). Chemotaxis was compared by determining the CFU of populations attracted to raffinose (c) (Dunn’s post hoc, p < 0.01). TTC data (a) represents three biological reps with three technical reps each. ATP data (b) represents two biological reps with three technical reps each. Chemotaxis data (c) represent three biological reps with three technical reps each.

To determine cell activity, TTC reduction and extracellular ATP per O.D. were quantified. E1 displayed higher respiratory activity (TTC reduction) than the lower three fractions (Figure 6a). Conversely, ATP levels per cell were lower in E1. This indicated that while E1 cells respired actively, they also consumed ATP generated more than the other fractions. To investigate the large number of chemotaxis related proteins in the lower fractions (Figure 3), we quantified swimming towards the chemoattractant Raffinose. E3 populations showed the highest chemotaxis with E1 the lowest.

### Phenotypes are not heritable

**Figure 7.**
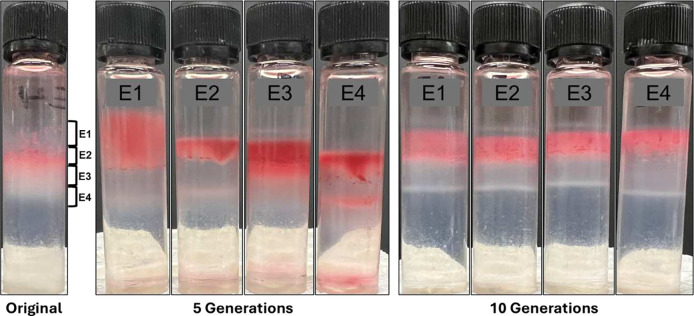
Heritability of phenotypes after 5 and 10 generations. Four fractions from the original exponential phase gradient were cultured for five and ten generations and subjected to Percoll density gradient centrifugation. Images are representative of experiments performed on two occasions.

To determine whether fraction specific phenotypes were retained through multiple cell divisions, we cultured fractions for five and ten generations. Gradient centrifugation of fractions after five generations yielded bands that overlapped with the respective original fractions (Figure 7). However, E1 included denser cells than the original fraction, indicating phenotypic changes in some of the cells. Likewise, fractions 3 and 4 also included zones with densities that differed to the original. Ten generations after separation into fractions, populations again included the full spectrum of buoyant densities. This indicated that fraction specific phenotypes were not retained during extended culturing, but that cells started diverging into multiple phenotypes again.

## Discussion

*B. diazoefficiens* can grow and maintain populations in soil and the rhizosphere, in addition to nodulating soybeans ^2^. Competitive fitness in soil requires adaptability to different niches, so that bet-hedging through heterogenous phenotypes would increase success of the species. We hypothesized that *B. diazoefficiens* USDA 110 displays phenotypic heterogeneity in phenotypes other than surface sugars. Density gradient centrifugation has previously been used to separate constituent phenotypes of an *E.coli* population, so we subjected various growth phase populations to Percoll^™^ density gradient centrifugation ^11^. The density gradient yielded many tightly spaced bands, confirming differences in buoyant density, and therefore phenotypic heterogeneity of populations. Fractions obtained upon density gradient separation showed differential binding to SBA lectin, which confirmed our previous findings ^17^. Proteomics revealed additional phenotypic differences among the fractions. Proteins associated with detectable phenotypes included polysaccharide synthesis, cell division, membrane and peptidoglycan synthesis, and chemotaxis, and we could confirm these differences in phenotypes among the various fractions. We also found differences in PHA content across fractions, as was reported for *Sinorhizobium meliloti* ^24^. These results show that *B. diazoefficiens* displays phenotypic heterogeneity in multiple phenotypes, and that each phenotype presents a spectrum that is significantly different across fractions.

### Dichotomy versus spectrum in heterogeneity

**Figure 8.**
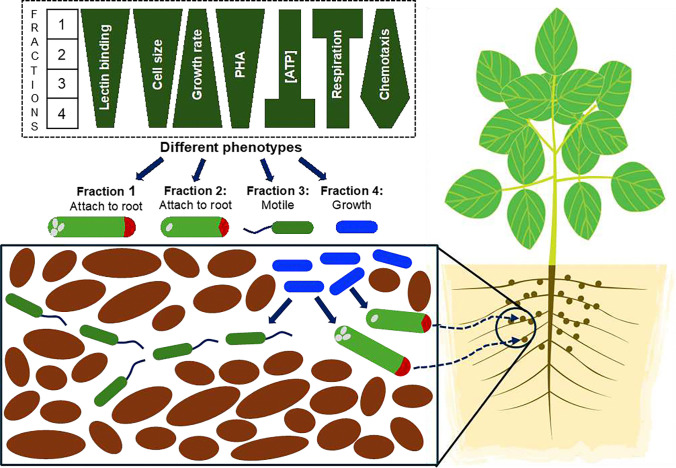
Proposed model for ecological fitness of *B. diazoefficiens* phenotypes. The box at the top shows differences in specific phenotypes across the four fractions. The phenotypes suggest that E1 cells are best prepared for nodulation, E3 cells for chemotaxis towards beneficial niches, and E4 cells for population growth in soil.

USDA 110 demonstrated a spectrum of heterogeneity in the various phenotypes tested. We observed a spectrum of cell sizes in the clonal population that separated into fractions (Figure 4a). The top fraction of stationary phase included exceptionally longer cells, but the lengths of lower fractions varied less. Fraction 4 cells divided the fastest while fractions 3, 2 and 1 had similar growth rates. We did not see two phenotypes but a spectrum in regard to presence of PHA, as proportions of PHA positive cells decreased from fractions 1 to 4. Lectin binding also displayed a spectrum since most of the cells in E1 and S1 bound to lectins and formed clusters, with lesser binding and clustering were seen in E2 and S2, and least binding in E4 and S4. Cellular energetics, including ATP per cell and respiratory activity also showed a spectrum, as did motility (Figure 8). Overall, all these phenotype spectrums indicate multiple sub populations. These spectra in phenotypes are in contrast to the established dichotomy in heterogeneity as first reported in 1976 for Lac operon expression in *E.coli* ^35^. Research in heterogeneity has focused on the presence or absence of a single phenotype in a homogenous population, viewing a phenotype dichotomously, as “A or B”. Examples of dichotomous heterogeneity include sporulation in *B. subtilis* where only some cells are primed to form endospores, giving the genus survival fitness in adverse conditions ^36^. A second dichotomous phenotype in *B. subtilis* is competence ^37^. A medically relevant example is flagellar expression in *Salmonella* ^5^. Virulence in some Salmonella is also heterogenous where some cells are virulent, whereas others take part in maintaining the population ^38^. Dichotomous heterogeneity in phenotype has been interpreted as bet-hedging ^39^. In contrast to dichotomous heterogeneity, some bacteria display a spectrum of heterogeneity. Nitrogenase in *Klebsiella oxytoca* is expressed heterogeneously, with individual cells having different levels of *nifHDK* mRNA as determined by RNA-FISH. This was ascribed to inherent stochasticity of transcription, likely through noise in the action of GlnK ^6^. Another example of spectral heterogeneity is *Streptomyces* where colonies are developmentally heterogenous, when within-colony phenotypic heterogeneity arises ^40^. This allows part of the population to survive multiple shifts in conditions, where the original phenotype would not support survival. Our results show that *B. diazoefficiens* displays heterogeneity on spectrums rather than dichotomously. *B. diazoefficiens* USDA 110 displayed a range of intensities for each of multiple phenotypes (Figure 8). This points to multiple subpopulations in culture growing under homogenous conditions. We propose that these ranges in multiple phenotypes position the culture for population success in the diverse conditions between bulk soil and soybean rhizosphere. While some cells should symbiotically associate with nodules, others are poised to persist in soils of varying nutrient and other properties. Thus, phenotypic heterogeneity would increase its survival under field conditions because of different input gradients, including concentration of nutrients in diverse environments like soil ^40^.

### Cell size variation

We found that *B. diazoefficiens* USDA 110 cells grown in PSY with arabinose were between 1 and 6 μm in length, in agreement with USDA 110 grown in yeast extract-mannitol medium ^41^. This differs from the paradigm for growth and cell division established in model bacteria such as *E. coli* and *B. subtilis* where cell length of daughter cells after division is identical. This is because peptidoglycan elongation synthesis in *E. coli* occurs across the cylindrical body of the rod, and septal synthesis occurs at midcell ^42^. Thus, exponential phase cell lengths vary by a factor of two when grown under defined conditions. Recent work on cell growth in *Alphaproteobacteria* indicates a different mechanism for cell elongation and division. The *Hyphomicrobiales* (*Agrobacterium tumefaciens, Brucella abortus*, and *Sinorhizobium meliloti*) display unipolar growth ^43^, as does *Caulobacter crescentus* ^44^. Polar incorporation of envelope components relies on homologous proteins shared in the *Hyphomicrobiales,* and distinct from the canonical elongasome. Peptidoglycan incorporation in *B. diazoefficiens USDA 110* was recently shown to occur unipolarly, like the other *Hyphomicrobiales* studied ^41^. This unipolar growth is due to incorporation of new peptidoglycan only at one of the cell poles ^45^, leading to unidirectional growth from the new pole generated after cell division. Stationary phase USDA 110 included cells twice as long as in exponential phase, as seen in E1. This too differs from the *E. coli* paradigm where stationary phase cells are smaller than exponential phase ^25^. These deviations from the model point to unique mechanisms of cell division and preparation for stationary phase. Collectively, these examples suggest that USDA 110 displays dimorphism, dividing asymmetrically, and yielding cells of varying sizes and phenotypes.

### Heritability of phenotypes

Individual fractions cultured for ten generations reverted to the full range of buoyant densities, indicating that buoyant density was not heritable. Even after five generations the density range of individual fractions expanded. The phenotype of specific fractions was not maintained across ten cell divisions. This suggests that the phenotype was not controlled through epigenetic imprints on individual genomes ^46^. Rather, phenotypic variation arose through cell division. Phenotypic heterogeneity can be due to regulatory mechanisms or to stochasticity where the outcome is random ^47 48^. Heterogeneity subject to regulatory mechanisms is termed bi-or multistability and should display repeatable behavior. The heterogenous phenotypes observed from homogenous culture conditions appeared repeatedly. This included buoyant density distribution, PHA, lectin binding profile, chemotaxis and cell activity. The repeatable appearance of these phenotypes points to underlying regulatory mechanisms rather than stochasticity in gene expression. Thus, the occurrence of multiple phenotypes under homogenous conditions in liquid medium indicates an established mechanism for heterogeneity in USDA 110. The species appears to have evolved to differentiate into different phenotypes during division.

### Proposed model

Growing *B. diazoefficiens* populations appear poised to divide into cells with different phenotypes. Various cells then have the capacity to occupy various niches depending on the functions they can perform. Phenotypic heterogeneity has been likened to bet-hedging where each cell type performs specific functions ^49^. This is beneficial to species occurring in complex or changing environments such as soil where physicochemical conditions vary. Resident microbiota consume available carbon, and growing root systems such as soybean introduce increased complexity through localized exudation of organic molecules that may act as nutrients or signals to bacteria ^50^. Depending on the locality *vis-à-vis* growing soybean roots, *Bradyrhizobium* encounter feast or famine. Roots provide organic carbon for growth and entry points for nodulation, but distal locations may lack growth-supporting carbon. The range of traits observed (Fig 8) may each benefit species’ success in some way. While some cells would symbiotically associate with nodules, others could be poised to persist in soils of varying nutrient and other properties. This would ensure that part of the progeny would succeed, irrespective of the set of local conditions. Larger cells with lectin-binding domains and reserve carbon in form of PHA (E1) are best poised for root adherence and ensuing nodulation. Smaller motile cells (E3) can translocate better through pores in soil, supporting dispersal of the species. Small faster growing cells, while not prepared for nodulation, divide faster contributing to population expansion (E4).

## Conclusions

Laboratory grown *Bradyrhizobium* comprised of sub populations with different phenotypes, that included cell size, PHA content, lectin binding profile, growth rate, cellular ATP, chemotaxis, and respiration activity. As these phenotypes play roles in population success in the heterogenous soil and soybean rhizosphere environments, we propose that heterogeneity helps slow growing *B. diazoefficiens* proliferate and maintain populations.

## Materials and methods

### Cultures and culture conditions

*B. diazoefficiens* USDA 110 was obtained from the Culture Collection of the Agricultural Research Service, United States Department of Agriculture. USDA 110 was tagged with GFP as described recently ^16^. *Bradyrhizobium diazoefficiens* spc 4 was obtained from Professor Hans-Martin Fischer (ETH, Zurich, Switzerland). *Bradyrhizobium diazoefficiens* USDA 110 and spc 4 were grown in PSY + arabinose as a carbon source, consisting of 300 mg KH_2_PO_4_; 300 mg Na_2_HPO_4_; 5 mg CaCl_2_·2H_2_O; 100 mg MgSO_4_·7H_2_O; 3 g peptone; 1 g yeast extract; 10 mg H_3_BO_3_; 1 mg ZnSO_4_·7H_2_O; 0.5 mg CuSO_4_·5H_2_O; 0.1 mg Na_2_MoO_4_·2H_2_O; 0.1 mg MnCl_2_·4H_2_O; 0.19 mg FeCl_3_; 1 mg thiamine-HCl; 1 mg biotin ; 1 mg Na-panthothenate; 1 g L-arabinose per liter. Growth while shaking at 30°C was quantified for 120 h by O.D. (600 nm). For some experiments chloramphenicol was added to avoid contamination as USDA 110 is resistant to chloramphenicol.

### Density gradient centrifugation and staining of fractions

Samples for density gradient centrifugation were taken at early exponential, mid exponential, transition, early stationary and late stationary phases (Figure 1b). Cells were harvested by centrifugation at 7,000 × g for 7 minutes at 4°C and resuspended in 500 μl sterile water. Percoll^™^ (Cat 17089101, Cytiva) was diluted with 0.15 M NaCl (with final NaCl concentration of 240 mM) and gradients were prepared in polycarbonate centrifuge tubes (Cat 355603, Beckman Coulter) by ultracentrifugation at 30,000 × g for 45 min (Beckman Optima Max MLA-55 rotor) at 4°C. Concentrated cell suspensions were laid on the top of preformed gradients and centrifuged for one hour at 30,000 × g at 4°C. To determine respiratory activity of cells, 10 ml liquid culture was pelleted at 7,000 × g for 7 min at 4°C and resuspended in 500 μl of 0.01% triphenyl tetrazolium chloride (TTC). The cells were left to stain for 45 min at room temperature before layering onto gradients. Tubes were imaged using an iPhone 13 camera. Cell viability was determined by using LIVE/DEAD^™^ Baclight ^™^ bacterial viability kit (Cat L13152, Invitrogen) to cell concentrates before centrifugation. Gradients were imaged using a stereo microscope with fluorescent 466/440 nm excitation and a 525/550 nm emission filter. In all cases, gradients were separated into four fractions for all further experiments as defined in Figure 1a. The fraction of lowest buoyant density (top fraction) was named fraction 1, and the highest buoyant density fraction was named fraction 4. Exponential phase fractions were named E1, E2, E3 and E4, and stationary phase were S1, S2, S3 and S4.

### Lectin staining

Exponential and stationary phase gradients were separated into four fractions (Figure 1a). To determine differential cell binding, lectin staining was performed on the individual samples with fluorescein-conjugated SBA (soybean agglutinin) as described ^17^. Samples were viewed by fluorescence microscopy using an Olympus BX53 Upright Compound Microscope with 466/440 nm excitation and a 525/550 nm emission filter and a 100-x oil immersion objective, captured using an Olympus DP70 digital camera. Image J ^51^ was used to create composite images. To determine the effect of prior lectin binding on separation in the gradient, cells were pre stained with lectins before centrifugation. Extracellular polysaccharide (EPS) was extracted, precipitated and quantified as described previously ^16^.

### Sample preparation for Proteomics

Exponential phase density gradients were separated into four fractions (Fig 1b), and fractions from four tubes were pooled. Percoll^™^ was removed from cells by filtering through polyvinylidene difluoride membranes with 0.2 μm pore size (Cat HVLP04700, Millipore). Percoll-free cells were obtained by cutting membranes into small pieces and transferring them into 15 ml conical tubes with 10 ml sterile water. Tubes were vortexed and manually shaken by hand to dislodge as many cells as possible from the membranes, filter pieces were removed using sterile tweezers, and cells were pelleted at 7,000 × g for 7 min at 4°C. Pelleted cells were resuspended in 450 μl sample treatment buffer and placed on ice. Cells were disrupted by applying 2 bursts of 30 s each of ultra-sonication, placing on ice for 20 s between and after bursts. The unbroken cells were removed by centrifugation for 2 min at 12,000 × g. The supernatant was transferred to clean tubes and stored at −20°C. Protein was quantified using Coomassie Protein Assay Reagent (Cat 1856209, Thermo Scientific) following the manufacturer’s instructions. The volume of sample required for a total of 1 μg of protein was calculated and transferred to a 1 ml Eppendorf, 20 μL DTT solution () and 1μl of loading dye was added and mixed by pipetting. Samples were loaded on Mini-PROTEAN TGX precast gels (Cat 4561094, BIO-RAD), resolving at 60 V for 30 min. Gels were stained using the Invitrogen Colloidal Blue Staining kit (Cat LC6025, Invitrogen), stained portions were excised and submitted to the Mass Spectrometry Facility at Carnegie Science, Stanford.

### Cell size determination

Exponential and stationary phase Percoll-free fractions of *B. diazoefficiens* USDA 110 (GFP) were prepared as for proteomics. Cells were harvested at 7,000 × g for 7 min at 4°C and resuspended in 3 ml sterile water. Drops of cell suspension were put on glass slides, covered with coverslip and the sides sealed with clear nail paint. Cells were imaged using an Olympus FV1200 Scanning Confocal microscope at 60X (oil immersion) using the GFP filter. Cell lengths were measured using ImageJ software ^51^. For this, all cells in three separate microscopic views were measured, with a minimum of 50 cells per fraction.

### Growth rate

Exponential phase fractions were prepared as described above. Undiluted, and fractions diluted by a factor of 20, 40 and 80, and 50 μl were added to 200μl of PSY media (+ Arabinose and chloramphenicol) in a 96 well plate (n = 16). Plates were incubated at 30°C in a FLUOstar Omega plate reader (BMG LABTECH), and the absorbance (600 nm) measured every 10 min for 1500 minutes. Data were imported into Microsoft Excel to plot growth curves, and the generation times derived.

### PHA analysis

To determine presence of PHA, Percoll-free GFP labelled cells were stained with Nile red (Cat 415711000 Thermo Scientific) at 0.1g/L and incubated for 30 min at room temperature. Cells were viewed using an Olympus FV1200 Scanning Confocal microscope using green and red filters with a 60 × (oil immersion). To visualize intracellular granules, exponential cells were washed twice using PBS (pH 7.4), resuspended in 5 ml EMC (100 mM sodium cacodylate, 2% glutaraldehyde, and 2% paraformaldehyde) and viewed by transmission electron microscopy at the Electron Microscopy Core Facility, University of Missouri.

To verify presence of PHA, cells were harvested, washed, and lysed in chloroform to selectively dissolve PHA and degrade the non-PHA cell components. An equal volume (v/v) of chilled ethanol (100%) was added to precipitate the PHA, followed by centrifugation and drying. The yield of PHA per gram of cells was calculated using the following equation.

$$PHA\;Yield=\frac{\mathrm{PHA}\;\mathrm{weight}\;(g)}{\mathrm{Cells}\;\mathrm{dryweight}\;(g)}$$

Fourier-transform infrared spectroscopy (FTIR) was used to analyze the functional groups in the sample structure, in a spectrum of 400 to 4000 cm^−1^ in the Agilent 600 FTIR spectrophotometer (Agilent Technologies, Santa Clara, CA, USA).

### Measuring cellular ATP and cellular respiration

#### Cellular ATP

Percoll-free cell fractions were diluted to the same O.D, 100ul transferred to 1 ml microcentrifuge tubes, and 2.5μl of 1.2M ice cold perchloric acid was added, mixed by vortexing for 10 s and put on ice for 15 min. The tubes were centrifuged at 30,000 × g for 7.5 min at 4°C and 250μl of 0.72M KOH and 0.16M KHCO_3_ was added. Tubes were vortexed and centrifuged at 30,000 × g for 10 min at 4°C. 100μl of supernatant was transferred to a polystyrene 96 well plate and 100μl of BacTiter- Glo Microbial Viability Assay reagent (Cat G8230, Promega) was added. The plate was put on a shaker for 5 min in the dark. Samples were transferred to a 96 well white plate and luminescence was measured using a luminometer (BioTek Synergy2 Microplate reader).

#### Cell respiration

Exponential cells of *B. diazoefficiens* USDA 110 (10 ml) were pelleted for 7 min at 7,000 × g. Pellets were resuspended in 500μl 0.01% TTC and left for 45 min. After density gradient centrifugation, four fractions of TTC stained cells were obtained and filtered to remove Percoll^™^ as explained above. All fractions were set at the same O.D. for normalization, and 5ml of each fraction was transferred into a 10 ml tube and centrifuged at 7,000 × g for 7 min at 4°C. The pellet was resuspended in 1 ml autoclaved millipore water. n-butanol (1 ml) was added and vortexed for 30 s. The suspension was centrifuged at 7,000 × g for 8 min at 4°C. The O.D. of the uppermost layer was measured at 490 nm. Non-TTC stained populations were used as blank.

### Chemotaxis

The chemotactic response of various fractions was determined using a capillary tube assay with raffinose as chemoattractant ^17^. Sterile capillary tubes with inner diameter of 1.1–1.2 mm, (Cat 22–260943, Fisher Scientific) were placed at an angle of 30°C in a petri plate containing P/10 base supplemented with raffinose (1 g/L). Water agar (3%) was used to seal the other end to prevent liquid escaping. After removal of Percoll^™^ as described above, the four fractions were resuspended in P/10 medium, set to the same O.D., and were put into wells of a 12-well plate. The open ends of the capillaries were dipped in cell suspensions in the 12-well plate at an angle of 45° to ensure that the open end is not blocked by the bottom of the plate but is submerged. Capillaries were left in the cell suspension for 60 min. The contents of the capillaries were expelled into microcentrifuge tubes by carefully breaking the plugged ends. Liquid remaining in the capillaries was withdrawn using a 100 μl tip. Cell counts were determined by plating serial dilutions onto R_2_A agar. To obtain enough sample volume, each technical replicate constituted three capillary tubes. Experiments were performed using three technical replicates and repeated on three separate occasions. Cell counts in P/10 without raffinose were determined and used as blank.

### Heritability of phenotypes

To determine whether phenotypes of fractions/subpopulations were maintained during cell division, the four fractions were inoculated into PSY + arabinose with chloramphenicol and incubated for 5 and 10 generations at 30°C, after which density gradients were performed again.

### Data analysis

Data were processed using Microsoft Excel, and statistical analyses were performed using PAST 4.03 ^52^, and Matlab ^53^. Violin plots were generated using Matlab ^53^.

## Data Availability

Raw data sets are available from the corresponding author.
